# Book Review

**Published:** 1904-06

**Authors:** 


**The Sanatorium Treatment of Consumption.** By T. N. Kelynack, M.D. Pp. 27. London: The Scientific Press Ltd. 1904.—This reprint from *The Hospital* contains the substance of a post-graduate lecture delivered at the Mount Vernon out-patient department. The author concludes as follows: "Sanatoria as remedial establishments have accomplished much, and will continue to bring benefit to many sufferers. As prophylactic institutions they may well be widely extended, and will be of incalculable service; but the chief benefit to the community will prove to be in their educational influence on the thought and life of the people. We do well to strive for cure, arrest, alleviation, but the ultimate goal must be prevention."

**Guy's Hospital Reports.** Vol. LVIII. London: J. & A. Churchill. 1904.—A notable contribution to this volume is the thesis for the M.D. Oxon of Dr. J. M. Fortescue-Brickdale, entitled "A Contribution to the History of the Intravenous Injection of Drugs"; together with "An Account of some Experiments in Animals with Antiseptics," and a bibliography. The author arrives at the following rather unsatisfactory conclusion: "With regard to the direct injection of antiseptics, the clinical evidence, open as it is to so many inevitable fallacies, gives at best only a very qualified support to the idea that such a proceeding can influence favourably the course of a bacterial disease; whilst the experimental evidence, considered as a whole, has hitherto been distinctly against it. The discovery of a drug of such selective capacity that, while injuring fatally the cells of living bacteria, it would leave the cells of the host entirely uninfluenced, seems at present a remote contingency, and until unquestionable experimental evidence of the existence of such a substance is forthcoming it would seem more rational to abandon further clinical trials, and thus to close another chapter in the history of the somewhat ill-fated *Chirurgia Infusoria*."

**The Physiology of Mastication and Kindred Studies.** By J. Sim Wallace, M.D., D.Sc., L.D.S. Pp. 32. London: J. & A. Churchill. 1903.—Dr. Sim Wallace's monographs are always worth studying, consequently we are glad that he gave us these three papers, originally read before the Odontological Society, within the covers of this book, conveniently arranged for consultation. The first essay explains most clearly the great importance and necessity for the thorough trituration, crushing and tearing of all food in the mouth, and well mixing the same with the saliva before swallowing it; and that when the food is of a fibrous character the more it is masticated the cleaner it will keep the teeth, and by thus being properly prepared before it is swallowed it will increase the facility of digestion. When it has entered the intestines, the fact of the food being fibrous cannot fail to assist the peristaltic action and facilitate the completion of the digestive process. The author points out that it is far better that we should eat whole-meal bread, and be content to swallow, after well masticating, the husk of the grain or bran, which inconsiderately is usually taken out of the flour in its preparation: the husk, being fibrous, assists mechanically the natural process of digestion. Moreover, starchy foods should not be swallowed too quickly, as the ptyaline of the saliva acts on it whilst in the mouth, and when it enters the stomach the action is almost *nil*. "It should also be remembered that water taken *after* meals not only tends to cleanse the mouth, but when it passes into the stomach it stimulates peristalsis and helps digestion." The essay on the feeding of children is well worth reading, also the writer's suggestions respecting the irregularities of the teeth, containing as it does many useful observations and hints. To sum up the general view, it is evident that the conclusions of the writer tend to show that the nearer a child is brought up and fed as in primitive times, the better the teeth will be, and the more perfectly the organs of digestion and the powers of assimilation of food will be developed.

**A Pocket-book of Clinical Methods.** By CHAS. H. MELLAND, M.D. Pp. vi., 88. Bristol: John Wright & Co. 1903.—This little book sets out the methods of performing the ordinary clinical tests. It is not nearly full enough in detail, and many of the methods are somewhat out of date. There is no mention of Widal's reaction, swabs from the throat, examination for thrush or tinea tonsurans. No mention is made of breaking up sputa with carbolic acid, which is so useful in some cases. We cannot recommend the book, it is too meagre.

**Practical Medicine.** Delhi (India): The Medico-Scientific Press.—This monthly record of the progress of medical science has now entered on its second year, and has achieved a distinguished success. From it we are pleased to learn that our own "articles in the December issue are unusually strong and worth many times the price."

**Asthma in Relation to the Nose.** By ALEXANDER FRANCIS, M.B. Pp. 136. London: Adlard & Son. 1903.—In this small book the author discusses the present views as to the etiology of asthma. He considers that from the results he has obtained from the treatment he advocates there is "in the asthmatic state a morbid connection between the nose and the respiratory centre which can so control or affect the respiratory centre and maintain it in such a state of instability as to allow otherwise trifling sensory irritations to provoke a reflex effect in the bronchial tubes." The most important part of the book is the treatment which the author advises, which is, under cocaine anæsthesia, to "draw a line on the septum nasi with a galvano-cautery point from a spot opposite to the middle turbinated body forwards and downwards for a distance of rather less than half an inch." In about a week's time the operation is repeated on the other side, and afterwards on alternate sides at intervals of ten days or a fortnight as occasion requires. A fresh spot is always selected to cauterise. The prognosis is best in those cases where there is no definite disease in the nose, and especially in the hereditary and purely "gastric" cases. When there are polypi the cauterising should be done before the removal of the polypi. The author has seen the condition made worse by the removal of these tumours. More than half the book is devoted to short notes on 402 cases treated by this method, and in which complete or great relief followed in the large majority. One is bound to admit that the results are most fascinating, and one only hopes that others adopting the same line of procedure may be equally happy in *their* results.

**A Guide to Urine Testing.** By MARK ROBINSON. Second Edition. Pp. 56. Bristol: John Wright & Co. 1904.—This very elementary book should be of use to these for whom it is intended—"nurses and others." Its existence implies that the work of urine testing is frequently left to the nurse. In hospital work when there are no clinical clerks or dressers it may be very convenient to utilise assistance of this kind, but unfortunately this practice leads to a further habit of expecting the urinalysis to be always done by others. This, we think, is a great mistake, and that it is unreasonable to expect our patients to bring with them a complete analysis made by some analytical chemist. The readiness with which these can now be obtained is no reason why the doctor should never use his own powers of observation, and such analysis must sometimes lead to unfortunate mistakes. Even with the assistance of this book there are many fallacies which would lead an unwary observer into error.

**The Johns Hopkins Hospital Reports.** Vol. X., Nos. 6, 7, 8, 9. Baltimore: The Johns Hopkins Press. 1902.—Four excellent papers are contained in this portion of the above Hospital Reports. Charles P. Emerson discusses "Metabolism in Albuminuria." The writer lays stress on slight rises of temperature and increase in the percentage of albumin as indicating the progress of nephritis. W. G. MacCallum contributes an article, with illustrative plates, on "The Regenerative Changes in the Liver after Acute Yellow Atrophy." Thomas McCrae and James F. Mitchell consider "The Surgical Features of Typhoid Fever" in a paper based upon the records of 275 cases of the disease treated in the Hospital in the two years between June, 1900 and 1902. Every case with surgical features is fully considered. A section on affections of the liver and gall-bladder associated with typhoid fever includes one case of abscess of the liver, successfully treated by operation, and five cases in which cholecystitis was suspected. Three of the latter recovered without operation, one ended in peritonitis following perforation of the gall-bladder, and one was successfully operated upon. One case of enteric ended fatally from acute appendicitis. Eight cases of perforation of the intestine are reported. Of the seven which were operated upon two recovered completely and a third lived a week and then died from toxæmia. With regard to the diagnosis of perforation, the authors write: "So great is the variation in the symptoms that no satisfactory general description can be given, nor is it well that it should be attempted. Those handling typhoid fever patients have to realise that perforation comes under many guises, and that to attempt its recognition from any constant combination of signs is hopeless." Two cases of suspected perforation were explored. One showed a mild grade of peritonitis, which cleared up after operation. In the other the symptoms were caused by intestinal hemorrhage. We have said enough to show that the paper we are considering is one which will repay the most attentive consideration. Finally, Benjamin R. Schenck has a paper on "The Symptoms, Diagnosis, and Surgical Treatment of Ureteral Calculus." The article is based on four cases of operation for this condition in the Johns Hopkins Hospital and on ninety-seven cases found in literature, and gives a good *resumé* of the subject.

Vol. XI., Nos. 1—9. 1903.—This number of these valuable reports contains an exhaustive study of pneumothorax, by Dr. Charles Emerson, which is discussed from the pathological, clinical, and experimental standpoints. Complete though the study is, we have been unable to pick out anything that can be described as new, but when it is desired to ascertain what is known about any special point in connection with pneumothorax this paper will remain for many years to come the work to which reference must be made. Another valuable paper is by Dr. Henry Cook upon "Clinical Observations on Blood Pressure." It is interesting to find that the value of strychnine as a stimulant to the vasomotor system can be proved by sphygmomanometric observations. Alcohol is said, however, to possess no power as a circulatory stimulant. The last paper is by Dr. Martin Tinker on "The Value of Tuberculin in Surgical Diagnosis." In the diagnosis of such diseases as tubercle of the kidneys, tuberculosis of the joints, and in the differentiation of Hodgkin's disease from tuberculosis of lymphatic glands, tuberculin appears to be valuable.

We need only add that these reports will contribute to uphold the fame of the institution from which they emanate.

**Elements of Surgical Diagnosis.** By A. Pearce Gould, M.S. Lond. Third Edition. Pp. viii., 607. London: Cassell and Company, Limited. 1903.—Works on surgery intended for students are becoming more and more divided into two classes, viz. those on principles and those on practice, or on diagnosis and treatment respectively. The work before us deals entirely with diagnosis, and must prove of great value to all who are commencing the study of surgery. The author has done well to separate the diagnosis of injuries from that of diseases. While this is not in strict accordance with the ways of nature, it has the merit of greater simplicity. The present edition contains chapters on "The Diagnosis of the Intracranial Complications of Middle-ear Disease," "The Diagnosis of Abdominal Tumours," and of certain acute abdominal diseases coming under the treatment of the surgeon, while frequent references are made to the application of bacteriology, skiagraphy, and blood examination to the art of surgery. The author has evidently studied condensation, for the book contains scarcely a superfluous word, but he has wisely avoided the opposite error of making it a mere notebook which would be irksome to the reader. It is written in pleasant and graceful language, and is in this respect a model of what such a book should be. No surgeon could read this book without adding materially to his knowledge of diagnosis, while the student will find it a mine of information on the art of acquiring ability to differentiate between various diseases and injuries.

**Transactions of the American Surgical Association.** Vol. XXI. Philadelphia: William J. Dorman. 1903.—This volume contains some excellent papers illustrative of the high degree of excellence in technique and operative ability attained by American surgeons. Some of the papers have already been published elsewhere, though this is not stated. With so much that is good, it is undesirable to comment on the relative merits of the different authors, but we have been chiefly interested in the articles on "The Surgery of the Simple Diseases of the Stomach," on "Prostatectomy and Galvanocaustic Prostatomy," and on "Intestinal Localisation." The volume covers a wide range of subjects, and should be read by those who are desirous of keeping abreast of modern surgery.

**Diseases of the Gall-bladder and Bile-ducts, including Gall stones.** By A. W. Mayo Robson, F.R.C.S., assisted by J. F. Dobson, M.S. Lond., F.R.C.S. Third Edition. Pp. xvi., 485. London: Baillière, Tindall & Cox. 1904.—The present volume differs widely from the first edition in the general arrangement, which is much improved, and in the treatment of the subject, from the increased experience of the author, whose knowledge of these diseases is of the highest order. It is a matter for congratulation to find that a large number of serious operations on the gall-bladder and ducts can be conducted with a comparatively small and diminishing mortality. The newest matter will be found on the operative treatment of gall stones, but the subjects as a whole are lucidly written, as they can only be by one whose knowledge is deep and whose experience is extensive. The illustrations are numerous, though perhaps not of the greatest artistic excellence. The work is to be regarded as the best English book on the operative treatment of the diseases under consideration, and it is likely to maintain its position for some years to come. At the end of the volume is placed an "appendix," which occupies a hundred and fifty pages without serving any very useful purpose. The accounts of the cases here given are too meagre to be helpful, and all that is valuable could be obtained by putting the operations and results in statistical form, which could be done in a few pages. There does not appear to us to be any sufficient reason for so largely adding to the size of the book by the inclusion of a list "of all the operations" that the author has performed on the gall-bladder and bile-ducts.

**The Imperfectly Descended Testis: Its Anatomy, Physiology and Pathology.** By W. McAdam Eccles. Pp. xi., 140. London: Baillière, Tindall & Cox. 1903.—The author discusses the subject of the title from all sides in a small and compact volume, which is copiously illustrated, mostly from original photographs. The chapter on "The Development and Descent of the Human Testis" gives merely a short review of the subject, and is provided with but one illustration. The student who wishes to fully understand this part of the subject must seek elucidation in the larger works on embryology. The other chapters are of a more practical character, and in them are discussed inflammation and tumour formation in the undescended testis, and the association of imperfect descent with the conditions of torsion of the spermatic cord, hernia, hydrocele, and failure of bodily growth and sexual development. In the appropriate place the indications for orchidopexy and directions for the performance of the operation are described. In a case of inguinal inclusion the parents, it is said, should be advised that an operation may ameliorate the condition, and cannot produce any harm or place the testis in any worse position than the existing one. The author is able to testify, from an inspection of "not a few cases" in which transplantation has been performed, that the testis has a very great tendency to mount again into the region of the superficial ring. Statistics which show the results of the operation as it affects the permanent position of the testis and its subsequent development are, as the author points out, wanting. We hope that some such will be collected and find a place in a future edition of this work. Taken as a whole the volume provides the reader with a good *resumé* of the present state of surgical knowledge and practice with respect to the undescended testis. It is written in the text-book style. No cases are recorded and no bibliography is appended. But little indication is given as to the part the author has taken in the development of this branch of surgery beyond collecting the statistics and the histological and other photographs which are here reproduced. The resulting illustrations are excellent.

**The Sterilisation of Urethral Instruments and their Use in some Urinary Complaints.** By Herbert T. Herring, M.B. Pp. xvi., 176. London: H. K. Lewis. 1903.—The author believes that the use of dirty urethral instruments causes the greater part of the distressing symptoms associated with urinary complaints, and that it is not impracticable for a patient who has to use urethral instruments to keep himself free from contamination if only he conscientiously practices the art of sterilising the instruments, and of using them after they have been sterilised, in the way described in the book before us. The methods given are those which the author has found efficient for the purposes mentioned. Ingenious contrivances for the sterilisation and lubrication of urethral instruments by boiling them in water and liquid paraffin are described and illustrated. Precise directions are then given as to the manner in which the patient shall use the instruments, and as to the antiseptic preparations he shall make beforehand. The endeavour has been to describe methods of sterilisation which are so simple that any person can learn them in a few lessons. Other parts of the book are devoted to a description and explanation of certain genito-urinary affections, especially those which may arise from a neglect of the principles of aseptic practice, the writer holding that it is for the best that patients should have some understanding of these matters. We have no hesitation in saying that if the methods advocated by the author were extensively adopted a vast amount of urinary infection would be avoided, and that we have heard of no better methods. But comparatively simple and practical as they are, we believe they will prove to be too troublesome and complicated for a considerable proportion of genito-urinary patients, certainly so for the out-patients of our hospitals. Amongst intelligent patients, however, the methods will have a wide field for useful employment. The account of them should be read by every practitioner who has heretofore been inclined to take the pessimistic view that self-catheterisation must end in infection, for he will acquire a hope of better things and will be encouraged to make more painstaking efforts to gain aseptic results, from which advantage will accrue to his patients and to himself. The lubrication of the rubber plugs employed, and of the outside of the glass tubes figured on page 34, is objectionable. Apparently in washing out the bladder a separate rubber bag is recommended for each four ounces of fluid used, a decidedly objectionable multiplication of instruments. We regret that a soluble lubricant is not provided for in the sterilisation scheme.

**Football Injuries.** By R. H. Anglin Whitelocke, M.D. Pp. 16. London: J. & A. Churchill. 1904.—A paper read before the Medical Officers of Schools Association on "Football Injuries," by Dr. R. H. Anglin Whitelocke, of Oxford, is well worth perusal, as the author has had exceptional advantages of studying and treating the individual cases from the very beginning, and has put the deductions drawn from his experience in a concise paper full of interesting detail. We readily endorse his opinion that Rugby football accidents are more common but less severe than those arising from the Association game, and are glad that he emphasises what we have in these pages pointed out, namely, the advisability of gradual training before the real work of a football season begins. One very interesting topic is the present method of dealing with sprains, and the author's condemnation of any splint or rigid apparatus. The details of his treatment are plainly put, and the rationale of each step given, and the results of such treatment are remarkable, twentyone days being the longest time that the player need expect to be kept from the football field. In another part of the paper Dr. Whitelocke gives a useful classification of the conditions that come under the head of internal derangement of the knee-joint. When a semilunar cartilage has to be removed he prefers a longitudinal to a transverse incision. There are many points in the paper which might give rise to some discussion, but to those of our readers who are connected with schools we may recommend the paper as one which is likely to be of considerable practical value.

**Drugs: Their Production, Preparation, and Properties.** By H. Wippell Gadd. Pp. xi., 180. London: Baillière, Tindall & Cox. 1904.—The first sentence of the preface expresses its nature very fairly: "A concise account of the principal vegetable, animal and chemical substances used in medicine." The book is divided into three sections, dealing successively with drugs of vegetable, animal and chemical origin, preceded by an introduction giving useful information as to methods of obtaining the soluble constituents, such as infusion, decoction, etc. The B.P. drugs are dealt with *seriatim*, the preparations of each being mentioned with their strengths. An example of the general up-to-dateness of the book is the mention under Kino of the fact of sterilisation of the tincture being necessary to destroy the enzyme which causes gelatinisation. A quotation from Robert Browning on the nature of Tragacanth is a novel application of poesy to pharmacy. Useful tables of weights and measures and alcohol strengths complete a work which should be of great use to the student of materia medica.

**The Therapeutics of Mineral Springs and Climates.** By I. Burney Yeo, M.D. Pp. viii., 760. London: Cassell & Company. 1904.—No one can be better qualified to write on any branch of therapeutics. This work is to some extent founded on the previous volume, entitled *Climates and Health Resorts*, a subject which has for many years engaged the attention of the author. The preface is written in a very humble spirit. It is pointed out that radio-activity may be the foundation of much of the magic virtue possessed by all mineral waters, but that "the time is not yet ripe for any positive, practical pronouncement on this subject." It would be an evil day for all mineral waters and watering-places if their prosperity were to depend on available chemical analysis and scientific evidence on the value of the waters. The utility of these places to the human family is such that we trust a scientific analysis of their value may be long deferred. It is quite pardonable to any hydrologist to magnify his office, and he naturally has a bias in favour of his own springs to the depreciation of all others. It has been a source of astonishment to the writer to observe how much may be done, and how great a variety of treatment may be devised, by variations in the mode of use of even one or two varieties of mineral water from different springs in the same town or village. Nearly five hundred pages are devoted to the study of mineral springs, and the information given appears to be very complete and reliable; then follows a chapter of nearly eighty pages on the application of mineral waters and baths to the alleviation and cure of disease, and here the sound common sense of the author is very obvious, for he does not accept any statement without criticism and the application of his general knowledge of the disease in question. Part II. is devoted to climate and climatic resorts, and here we are sorry to observe that Clifton is no longer included as one of the health resorts of this country. A short chapter on "Treatment in Sanatoria," and a list of those well established, adds to the utility of the volume.

**Dispensing Made Easy.** By WM. G. SUTHERLAND, M.B. Pp. vii., 102. Bristol: John Wright & Co. 1904.—The title of this book brings to mind the title of another work, *Killing no Murder*. We hope that the motive of the general practitioner mentioned in the preface, namely, to make dispensing "as easy as possible" is by no means the chief point considered in his surgery. It is interesting to note in the introduction that according to the author the methods of the dispensing chemist cannot be adopted by the medical practitioner in his limited time, and also that the reason is that systematic methods, solutions of salts, etc., are used by the latter, and therefore, arguing by inference, are not by the former. We had fondly imagined till we read this work that if system were carried out anywhere it would be in the pharmacy to which we entrusted the compounding of our patients' prescriptions. To proceed from the general to the particular, we are glad to notice that a poison cupboard for potent preparations *and* such preparations as are affected by light, santonine being particularly mentioned, —one cannot help recalling a particularly regrettable incident that occurred a short time ago through santonine being mistaken for strychnine and the latter dispensed in error, the two bottles being placed side by side—should certainly be used. Further, however, we are told not to keep our liq. strychnine, liq. arsenic, or liq. morph. in the poison cupboard, as "there is no advantage in keeping *all* poisons (the emphasis is the author's) in the poison cupboard, etc." Further comment is needless. Under a prominent head-line of "How to Cut Down the Drug Bill without Loss of Therapeutic Efficiency," we meet with the surprising statements that "aqueous" tinctures should be used, that they are pharmaceutically better than B.P. tinctures. We wonder how many drugs lend themselves to this process. Perhaps the feature of the book most to be deprecated is the way in which in his formulæ the author departs altogether from the British Pharmacopœia, a work that with infinite labour is put together by a representative council of medical men, aided by the first pharmacists of the country, as a standard for the preparation and combination of drugs, and in its place are suggested purely unofficial preparations made by an American drug house. No feature of present-day prescribing is more lamentable than the tendency to prescribe, not from the B.P., but from the price list and specious advertisements of various drug houses—chiefly American and German. This author throughout booms one particular American firm, whose pamphlets and so-called "specialities" flood our tables and waste-paper baskets. Ince's dispensing aphorisms are a welcome relief, but surely never did the veteran pharmacist's wise mottoes for the dispensing counter appear in such strange surroundings. Throughout the principle appears to be, not to carefully prepare your solutions, but to take So-and-So's tablets and dissolve so many in so much water. Liquid extracts of gentian, bitter orange, squills, rhubarb, hyoscyamus, are examples of refinements (?) of pharmacy of which the B.P. is innocent, but which are necessary for "easy dispensing," and for which presumably we must go to our drug house "across the water." The one thought that arises when we read this book is, that if the author is right, the sooner we adopt the German principle of leaving all dispensing in the hands of the chemist, the better. In view of a recent occurrence in an institution in the South of England, accuracy and not easiness needs to be the motto of the dispenser, whether medical or pharmaceutical.

**Manual of Midwifery.** By W. E. Fothergill, M.D. Third Edition. Pp. xviii., 506. Edinburgh: W. F. Clay. 1903.—This book had already become deservedly popular before it reached the present edition. Although the subject is classified carefully it is pleasant reading and the descriptions are clear. The subject of development has been brought into line with the recent work which has been done in this branch by Hart, Yalland, Webster, Foulis and others. For instance, the anatomical space so long described as an outfolding of the somatopleure, is now shown to be a cleavage at an earlier date in the epiblast, and to have, therefore, in its formation no mesoblast. The early ova of Spee, Leopold and Peters are figured and described; they illustrate very plainly some of the earlier changes of the embryo. The chapter on "Forceps" is excellent, much of the careful work on the subject by Milne Murray being extensively acknowledged. The diseases of the puerperium have not sufficient space devoted to them, the details of management being very scanty. This subject is of great importance in midwifery, and is too often dismissed with but few words. The illustrations throughout are good, and some of the diagrams especially useful. We can recommend the book as concise and yet pleasant reading both to students and practitioners.

**The Transactions of the Edinburgh Obstetrical Society.** Vol. XXVIII. Session 1902—1903. Edinburgh: Oliver and Boyd. 1903.—This is an interesting volume, full of good papers. The value of Bossi's dilator is testified to by Dr. Frost and Professor A. R. Simpson in two cases of eclampsia. It seems to have acted well, dilating, without damaging the uterus, in about half an hour, even when the woman was not in labour, and not to have damaged the cervix. They claim that it acts not merely mechanically, but also by stimulating the uterine contractions. The most interesting paper is by Dr. Berry Hart on "Hydatidiform Mole and its Relation to Deciduoma Malignum." After discussing the histology of the normal villus with special reference to Langhan's layer and the syncytium, both of which he considers fœtal, he points out that it is in this region the change in hydatidiform mole takes place, and also from these cells that deciduoma malignum arises, thus accounting for the fact that the latter has so often been recorded as following the former. He then advances the theory that the overgrowth of the villus in these moles is the continuation of the natural process of growth in the villi, which is usually checked by fœtal thyroid secretion. When the embryo dies early this action is not present and hence the disease. He is not clear why this should take place so rarely, as early death of the fœtus is comparatively common. Dr. Laskir gives a number of cases of pyrexia following labour which he considers to be non-septic. With most of these we cannot agree. He asks for a reason why a woman should have a rigor after washing out the uterus, which four of the cases had. Surely that may be taken as almost if not quite conclusive evidence of intra-uterine sepsis. Two papers on "The Treatment of Threatened Eclampsia by Thyroid Extract" do not show that it has much value. Most of the other papers refer to the technique or results of abdominal operations.

**Transactions of the Ophthalmological Society of the United Kingdom.** Vols. XXII. and XXIII. London: J. & A. Churchill. —Having had the pleasure of perusing these volumes we have nothing but praise to offer with regard to them. They are well got up, and the illustrations, both in black and white and in colours, leave nothing to be desired. These illustrations, which are always a feature of the *Transactions*, are very numerous, and no expense has been spared in their production; indeed, when one thinks of their cost one almost feels inclined to call it a "prodigality of illustrations." The text, too, contains a perfect feast of interesting matter. In Vol. XXII. may be found the entire Bowman Lecture for the year on "Keratitis," delivered by Professor Fuchs, of Vienna, before the Society; while Vol. XXIII. has for its *pièce de résistance* a most exhaustive paper by Dr. Louis Werner, of Dublin, on "Intra-ocular Echinococcus with Brood Capsules." Altogether the two volumes form an extremely interesting collection of rare and abnormal cases, and the committee are to be congratulated on their selection and the wealth of material they had to select from.

**The British Journal of Children's Diseases.** Vol. I., No. 1. January, 1904. London: Adlard & Son.—This new journal contains some interesting and valuable articles. It has been customary to consider that there is little difference between disease in children and in adults. Too much may perhaps be made of diseases of children as a speciality, but the more one studies ailments in children the more one finds how much there is that is not to be found in general text-books upon medicine. At the same time, we think that most works upon children's diseases are very unsatisfactory. When facts of importance are mentioned they are too often hidden amongst a mass of information that can be gleaned elsewhere. If the *British Journal of Children's Diseases* maintains the standard of the first number we think it will be a useful addition to medical literature.

**Transactions of the American Dermatological Association.** May, 1903. New York: The Grafton Press.—Papers of great interest in a readable form are presented in this volume from the pens of well-known dermatologists. It is of considerable practical interest to see how large a part phototherapy and radiotherapy play in these reports. Three papers are worthy of notice. Stelwagon's steady, self-respecting and critical essay, involving, as it does, the record of his own experience, can only prove of service to those who are anxious to assign the proper position to the X-rays in the treatment of cutaneous disorders. His allusion to medical hysterics, and his rather caustic remarks on the unbalanced optimist does not prevent him from reciting a fairly long list of diseases in which he has found the treatment of great use. Pusey, whose name also is well known in the world of photo-therapeutics, contributes a paper on the same subject, and does a good deal to deliver the question from the region of absolute empiricism. Montgomery, in his article on "The Present Status of Phototherapy," practically reviews the condition as at present date it obtains at Copenhagen. In company with many who have used the smaller forms of lamps, he is not altogether persuaded of their inefficiency. His collated remarks at the end on the penetration of light from different sources through layers of skin, etc., are of scientific interest. Among the other papers may be mentioned one from Van Harlingen, who attempts to make out a case for gangrenous cutaneous affections being of hysterical origin, though being without injury, accidental or self-inflicted. Also Morrow's criticism on "Syphilis and the Medical Secret." In this paper he reviews the question of the responsibility of the profession with regard to the patient, and the latter's relation to others and to the State. His suggestion that legislation might compel the revelation to the sanitary authority in the case of anyone engaged in the occupation of nurse, domestic baker, waiter, barber, vendor of toys and food stuffs, or concerned in the preparation of food or drink, demands careful thought, tending, as it does, to protect the innocent from so terrible an infection.

**Modern Nursing in Private Practice.** By Sir William Bennett, K.C.V.O., F.R.O.S. Pp. 23. London: The Scientific Press Ltd. 1904.—This address was delivered to nurses at St. George's Hospital; it contains most excellent advice and some pertinent home truths which deserve to be widely read. Sir William Bennett has had considerable experiences of private nurses, good and bad, and he is able to offer some excellent suggestions to nurses as to what they should avoid. We heartily endorse his opinions and recommend them to all concerned.

**The Pocket Therapist: A Dictionary of Disease and its Treatment.** By Thomas Stretch Dowse, M.D. Pp. 411. Bristol: John Wright & Co. 1903.—This little volume may be looked upon as a pocket dictionary of therapeutics. The arrangement of the subject-matter is alphabetical. For ready reference and as an aid to memory we can cordially recommend the book. The directions for the treatment of disease are condensed, but the condensed matter is remarkably good and thoroughly modern, and in our opinion cannot fail to frequently help the reader.

**Serum Therapy.** By R. T. Hewlett, M.D. Pp. viii., 262. London: J. and A. Churchill. 1903.—The treatment of disease by antitoxins and sera has now become so general that a short work dealing with their preparation, standardisation and method of administration will be readily appreciated by all students of medicine. An instance has recently come to our notice where diphtheria antitoxin has been given by the mouth, and yet another where antistreptococcus serum has been administered in an infection due to an entirely different organism. A perusal of Prof. Hewlett's book would do much to correct such errors, and will afford guidance in the selection of suitable cases in determining the dosage, the frequency of administration, and estimating the probabilities of success. The true value of serum-therapy cannot be gauged until the principles governing its administration are understood by the profession, and for this reason we heartily welcome Professor Hewlett's work.

**A Manual of Pathology.** By Joseph Coats, M.D. Fifth Edition, revised throughout by L. R. Sutherland, M.B. Pp. xxvi., 1287. London: Longmans, Green & Co. 1903.—The fifth edition of this well-known text-book opens with a prefatory notice of the late Professor Coats by Sir W. T. Gairdner. The present edition has been revised throughout and edited by Professor L. R. Sutherland, and "may be said to have had the counsel and support of the late author up to the very close of his life." Although lapse of time and consequent advance in knowledge, and sometimes change of view, have made alterations necessary, the general plan and scope of the work have been carefully and successfully preserved. Thus the book will be found to present the same general features which have secured its success in the past, but improved and brought up to the knowledge of the time. The section on bacteriology has been omitted; this was never a strong point of the work, and the editor is no doubt right in saying that it can now only be dealt with in special treatises. As a trustworthy guide to morbid anatomy the book has few equals, and no superior in the number, variety, and excellence of its illustrations. In the present issue these have been increased from 490 to 729, they form an admirable series, have been beautifully reproduced, and by themselves alone make the book a valuable possession. This fifth edition seems to promise a prolonged life of prosperity to the volume, which in itself is a noble monument to the arduous labours of a lifetime.

**Cancer and Pre-cancerous Changes: their Origin and Treatment.** By G. H. Fink. Pp. 105. London: H. K. Lewis. 1903.—This book contains an account of various views as to the origin, prevention and treatment of cancer. The author seems himself to have adopted the parasitic theory, and most of his observations are based on that. It is not written to maintain any original view, nor does it contain, so far as we have observed, any new evidence. The author says his object in writing the book "is to deal as consistently as possible with the probabilities in the life history and development of cancer, so far as their knowledge at the present time admits." What this exactly means it is difficult to see. Major Fink tells us how he first forwarded a paper on cancer to the India Office, and how this was referred to the Cancer Research Committee. We have unfortunately to wait until the next edition of the book to know whether this Committee finally decide to aid Major Fink in investigating the subject. Why are we told all this? What possible bearing can it have on the contents of the book, except to show that his own investigation has very little, if anything, to do with it at present? A great deal of the book seems to us to read like a work of the imagination. No doubt if Major Fink carries out any real investigation he may have something original, and based on evidence, to put before the profession later on. We also hope by that time he will have learned that fewer words and a clearer expression of his meaning will greatly assist the reader. We may give an illustration of the way in wichh the author allows his imagination play. He first tells us that one of the most settled points with regard to cancer is "that there is a loss of nervous control, attended with strong emotional feeling, previous to the local manifestation of cancer." And he goes on to say that ten per cent. of young people in a train in London may be observed to have chorea. He then argues that chorea in youth causes cancer in late adult life, and refers us to the long incubation period in hydrophobia as an analogous condition, and explains the onset of cancer so late by suggesting that there has been for many years an "absence of the explosive cause which sets free the reins of control." Here, indeed, is a lucid expression of a view, founded on overwhelming evidence! We cannot help thinking that the author has been dreaming about cancer, and has confused the impressions of his sleeping hours with actual facts.

**Studies from the Department of Pathology of the College of Physicians and Surgeons, Columbia University, N.Y.** Vol. VIII. For the Collegiate Year, 1901–2. [Reprints.].—The director of this department, Prof. T. Mitchell-Prudden, has grouped together in this volume the reprints of the more important studies published by workers in the department in various medical journals during the years 1901–2. This is certainly a convenient method of collecting together papers too valuable to be buried in the bound volumes of current periodicals, and by facilitating reference should help other workers who are investigating the subjects dealt with here. Sixteen papers are presented, and without enumerating their titles we may say that they will all repay perusal, and refer our readers to the volume itself. Seven of the papers deal with bacteriological subjects.

**A Practical Handbook of Midwifery.** By Francis W. Nicol Haultain, M.D. Second Edition. Pp. viii., 253. London: The Scientific Press, Limited. [1902.].—This book gives within a small compass the chief points of practical importance in the study of obstetrics, and the present edition has been considerably enlarged and revised. In chapter iii. no mention is made of the probable toxic origin of uncontrollable vomiting, a theory which during recent years has found considerable favour. In the treatment of eclampsia the hypodermic injection of pilocarpine is recommended, but the danger of giving this drug when the patient is unconscious is overlooked. The low mortality of Cæsarian section at the present time is causing that operation to take the place of craniotomy, and the author thinks that in cases where the child is living the latter operation will soon be regarded as obsolete. The last chapter is devoted to the management of the infant, including the artificial feeding. The book is one we can recommend both to students and practitioners.

**A Handbook of the Diseases of the Eye and their Treatment.** By Henry R. Swanzy, M.B. Eighth Edition. Pp. xix., 678. London: H. K. Lewis. 1903.—We have received the eighth edition of this work, which so closely follows the seventh as to be almost like a journal of ophthalmology. It must be very gratifying to the author to find so great a demand for his work. At this we are not surprised, as for what might be called a small book on ophthalmic surgery, this compilation is still without a peer. To the ordinary student of medicine, who nowadays has so many subjects to claim his attention, we recommend the work strongly. The busy practitioner who comes across a puzzling eye case now and then will find in its pages the very latest information, and that given in such a concise and clear form as to render his search after knowledge in this direction a comparatively easy task. All the recent advances in ophthalmic work are set out at large, several of the chapters having been re-written, and the whole forms a work which does the author the greatest credit.

**Ophthalmic Nursing.** By SYDNEY STEPHENSON, M.B. Second Edition. Pp. xiii., 191. London: The Scientific Press, Limited. [1902.]—The second edition of Sydney Stephenson's *Ophthalmic Nursing* has now appeared, and we can congratulate him on the rapid sale the first edition has had. This shows how much his book is appreciated by the nursing fraternity. He points out in his preface that the second edition brings the text up to date with regard to new drugs and modern methods of sterilisation without increasing its bulk to any appreciable extent.

**Golden Rules of Refraction.** By ERNEST E. MADDOX, M.D. Pp. 86. Bristol: John Wright & Co. [N.D.]—This little volume is much more than a mere collection of aphorisms culled from a text-book on refraction. It is wonderfully concise, comprehensive, clear, and well arranged. Not only does it contain all the more important facts relating to refraction *in a readable form*, but there is also a good deal of information of a practical nature about the uses and advantages of the various kinds of apparatus required, and of the sorts of lenses and spectacle frames best suited for various conditions.

**Sight and Hearing in Childhood.** By ROBERT BRUDENELL CARTER and ARTHUR H. CHEATLE. Pp. viii., 120. London: The Scientific Press Ltd.—This little book is written to explain to parents and teachers the importance of the care of the sight and hearing of children under their care. In as clear and popular way as possible the mechanism of sight and hearing is explained, as also the common causes of defective vision and deafness, with some of their more obvious symptoms. The likely results of these defects are pointed out and the indications for treatment explained. This excellent little book should be in the hands of all teachers.

**Lessons in Disinfection and Sterilisation.** By F. W. ANDREWES, M.D. Pp. 222. London: J. & A. Churchill. 1903.—This little book, containing as it does some elementary teaching on the subject of bacteriology, will be found a useful guide to students and nurses in matters relating to disinfection. The information is not couched in language of too technical a nature, and can be readily understood by persons who have not received a medical education.

**Lessons on Massage.** By MARGARET D. PALMER. Second Edition. Pp. xvi., 261. London: Baillière, Tindall & Cox. 1903.—To this second edition has been added a chapter on the

Schott treatment of chronic diseases of the heart, describing the various exercises used for this purpose at Bad Nauheim. Besides this, a chapter on bandaging will add to the value of the book.

**"First Aid" to the Injured and Sick.** By F. J. Warwick, M.B., and A. C. Tunstall, M.D. Third and Revised Edition. Pp. xiii., 236. Bristol: John Wright & Co. 1903.—There is but little to comment on in this small volume. It contains in the first part a short survey of the anatomy and physiology of the body, and in the second chapters bandaging, hemorrhage, wounds, fits, &c. The diagrams are good, and several new ones have been added. There is a very useful little chapter on preparations for the reception of a case of accident and sudden illness. The work is intended as an advanced ambulance handbook, and it performs this function very well.

**Annual Report of the Smithsonian Institution for the year ending June 30th, 1902.** Washington: Government Printing Office. 1903.—This is not a medical volume; nevertheless, it contains much of scientific, and therefore of medical interest. Such topics as those of Becquerel on the Radio-activity of Matter, and Bateson on the Problems of Heredity and their Solution, are popular subjects of the time, whilst Professor Dastre's essay on the Life of Matter forms a striking contrast to the mutability of mundane things as shown by Russell's account of the volcanic eruptions of Martinique and St. Vincent.

**The Edinburgh Medical Journal.** New Series. Vol. XIV. Edinburgh and London: Young J. Pentland. 1903.—The original articles on a variety of medical topics are of more than the usual importance, but Dr. Sutherland's Glimpses of the Madrid Congress and Southern Spain forms a story which will be read with profound interest by those who visited the Iberian Peninsula last year, and by those who propose to attend the International Medical Congress at Lisbon in 1906. The introductory address to the Class of Anatomy in the University of Edinburgh, by Professor D. J. Cunningham, and the valedictory address to the Medico-Chirurgical Society of Edinburgh, by Professor Sir Thomas R. Fraser, are of more than ephemeral interest.

**The Prevention of Consumption.** By Alfred Hillier, M.D. Pp. xv., 226. London: Longmans, Green & Co. 1903.—The author, as the secretary to the National Association for the Prevention of Consumption (London), is entitled to write with all authority, and a preface by Professor R. Koch states that "the book in all respects represents the latest scientific views, which are so clearly expounded that every intelligent reader can derive reliable instruction from them. . . . The more readers the book finds the more good it will do." The preface contains the following statement: "Tuberculosis may be cured, can be avoided, and ought to be prevented." The book proceeds to show how these two latter feats are to be accomplished: the clinical and therapeutic aspects of the subject are not included. The chapters on Infection and Personal Precautions should do much in aid of the objects of the National Association.

**A Handbook of the Open-air Treatment, and Life in an Open-air Sanatorium.** By Dr. Charles Reinhardt and Dr. David Thomson. Second Edition. Pp. xv., 176. London: John Bale, Sons and Danielsson, Limited. 1902.—This concise account of the modern treatment of consumption and other tuberculous diseases, with descriptions of the open-air sanatoria in the British Isles, should be very useful in educating the profession and the public in the work which the National Association is endeavouring to do. The authors are both connected with the Stourfield Park Sanatorium at Bournemouth, and one of them also with the Hailey Sanatorium at Wallingford. The book is profusely illustrated, and interleaved with pages of advertisements, which were better omitted from any future editions which are likely to be required, when some notice of the London Sanatorium at Pinewood, Wokingham, might well be included.

**Muco-membranous Entero-colitis.** By Maurice de Langenhagen, M.D. Pp. vi., 115. London: J. and A. Churchill. 1903.—The author, a consulting physician at Plombières, Vosges, classes this malady as one of the manifestations of the arthritic diathesis, and he describes it as due to a superficial catarrh of the mucous membrane of the large intestine, caused in the first instance and afterwards kept up by coprostasis. He considers it to be intimately associated with Glenard's malady, enteroptosis having co-existed in all advanced degrees of the disease. Another usual complication is intestinal lithiasis, and possibly also appendicitis. All muco-membranous enteritic patients are neurotics, and not unfrequently uterine troubles completely mask the intestinal affection. The usual length of the illness seems to be about three years, but this may be considerably abridged by treatment, or on the other hand it may be extended to twenty or thirty years. The hydro-mineral treatment at Plombières, combined with regimen and evacuating medicines, has given excellent results.

**A Practical Text-book of the Diseases of Women.** By Arthur H. N. Lewers, M.D. Sixth Edition. Pp. xviii., 533. London: H. K. Lewis. 1903.—The present edition of this excellent manual has been thoroughly revised and brought up to date. The first chapter, describing the proper method of investigating a case, is of great practical value to the student. With regard to uterine fibroids, the author says that of operative procedures hysterectomy holds the first place. He recommends supra-vaginal amputation of the body with intra-peritoneal treatment of the stump whenever possible, and panhysterectomy for those cases in which the tumour involves the cervix. Oöphorectomy he considers obsolete. Malignant disease of the cervix may, he thinks, in some cases be treated by supra-vaginal amputation of the cervix, and he says "that in most cases this operation, properly performed, gives the patient just as good a chance of non-recurrence as vaginal hysterectomy." A full description is given of the supra-vaginal operation, and a number of cases are recorded. We think, however, that many of these would by most operators be treated by the more radical method of vaginal hysterectomy. The book, which is well illustrated and printed in good type, affords an excellent introduction to the study of gynæcology.

**Transactions of the American Pediatric Society.** Vol. XIV. New York: Reprinted from *The Archives of Pediatrics*. 1902.—There is nothing of unusually noteworthy interest in this volume. As is usual in American journals of children's diseases, the management of milk-feeding is the subject of several papers. There is one on fat percentages by Dr. Westcott, another on the proteids of milk by Dr. Dwight Chapin, and a third also by Dr. Chapin on the place of cereals in infant feeding. The remarks of Dr. Griffith in the discussion which followed the last paper seem to us to be of interest:—"In cow's milk we are dealing with a substance which you can never make just like human milk. It is beyond the possible. Therefore there can be no absolutely right way in which we must treat it before it is given to a child since, after all, it is an unnatural food, and any mixtures made with it are at best but approximations to, and imitations of, the natural article." He further adds:—"In the first place, then, we are dealing with an unnatural milk, and no absolute rule can apply to it. A second reason why there is no rule of feeding which can be called absolutely right is that we are dealing with babies as individuals." Various cases of interest are recorded, and this volume contains a valuable index of all the volumes (fourteen in number) published by the Society.

**Polyphase Currents in Electro-therapy.** By GEORGE HERSCHELL, M.D. Pp. 44. London: Henry J. Glaisher. 1903.—This is an account of the application to therapeutic purposes of a variety of the common alternating current. In the latter the force gradually increases to a maximum, as regularly declines, and then passes through the same curve, but in a negative sense. In the triphase system there are at any moment three such currents, each of which is at a point of the curve equally distant from the other two. Though their algebraic sum is zero, they produce a rotating magnetic field or electrical whirlpool in the tissues acted upon by them, and it is claimed that this form of electricity has a practical therapeutic value. Dr. Herschell, finding that it was impossible to regulate sufficiently the currents obtained from ordinary rotary converters, has brought out an apparatus which gives when needed a high E.M.F. with slow phase of alternation. Thus he can develop a current of rapid alternations which acts on the metabolism of the body, or a slow one which causes muscular contractions, especially in unstriped muscles. The generator can be driven from a 100 volt continuous street current or from a 12 volt accumulator, and the current applied by baths or electrodes which are three in number. He claims that these currents cause: (1) an increase in the tension of the pulse and in its amplitude; (2) a great rise in the excretion of urea; and (3) an immediate action on the movement of the gastro-intestinal tract. The length of stay in the stomach of a test meal is reduced, and an action of the bowels is often produced by the application of the current then and there. Certainly a battery which without drugs or enemata causes immediate defæcation, and in obstinate constipation leads to a final cure needs no recommendation. Its value in nervous dyspepsia and neurasthenia with a low tension pulse may be forgotten, but immortal fame awaits this invention if it only lives up to its early promise in the removal of constipation.

**A Pocket Dictionary of Hygiene.** By C. T. Kingzett and D. Homfray. Second Edition. Pp. 112. London: Baillière, Tindall and Cox. 1904.—This small volume gives an explanation of the terms used in sanitary science, and supplies in a condensed form much information relating thereto. It should prove serviceable to medical officers of health and sanitary inspectors.

**The Examination of the Urine.** By J. K. Watson, M.D. Pp. 30. London: The Scientific Press, Limited.—A small book intended solely for the use of nurses dealing with the physiology and pathology of the urine and its examination so far as the subject appertains to the province of the nurse. The first two parts, on the *Urinary apparatus and the characters of urine in health and disease*, are well arranged and easily followed; the chapter on the *Methods of examination* is by no means so good, and will probably be found difficult to understand by anyone not well versed in chemistry; too much explanation is attempted and too little is explained. The various tests are not particularly clearly laid down, and fallacies are repeatedly hinted at without suggestions being given to exclude them. An attempt is made at the end of the book to tabulate the condition of urine corresponding with some of the commoner diseases; where this table is intelligible it becomes misleading. On the whole, nurses might read less and know more than this volume offers.

